# Study of Kinetic Freeze-Out Parameters as a Function of Rapidity in pp Collisions at CERN SPS Energies

**DOI:** 10.3390/e23101363

**Published:** 2021-10-19

**Authors:** Muhammad Waqas, Huai-Min Chen, Guang-Xiong Peng, Abd Al Karim Haj Ismail, Muhammad Ajaz, Zafar Wazir, Ramoona Shehzadi, Sabiha Jamal, Atef AbdelKader

**Affiliations:** 1 School of Nuclear Science and Technology, University of Chinese Academy of Sciences, Beijing 100049, China or waqas_phy313@ucas.ac.cn (M.W.); chenhuaimin17@mails.ucas.ac.cn (H.-M.C.); 2 Department of Mathematics and Science, Ajman University, Ajman P.O. Box 346, United Arab Emirates; a.abdelkader@ajman.ac.ae; 3 Nonlinear Dynamics Research Center (NDRC), Ajman University, Ajman P.O. Box 346, United Arab Emirates; 4 Department of Physics, Abdul Wali Khan University Mardan, Mardan 23200, Pakistan; ajaz@awkum.edu.pk (M.A.); sabihajamal12@awkum.edu.pk (S.J.); 5 Department of Physics, Ghazi University, Dera Ghazi Khan 32200, Pakistan; zwazir@gudgk.edu.pk; 6 Department of Physics, University of the Punjab, Lahore 54590, Pakistan; ramoona.physics@pu.edu.pk

**Keywords:** rapidity, transverse momentum spectra, kinetic freeze-out temperature, transverse flow velocity, kinetic freeze-out volume, 12.40.Ee, 13.85.Hd, 25.75.Ag, 25.75.Dw, 24.10.Pa

## Abstract

We used the blast wave model with the Boltzmann–Gibbs statistics and analyzed the experimental data measured by the NA61/SHINE Collaboration in inelastic (INEL) proton–proton collisions at different rapidity slices at different center-of-mass energies. The particles used in this study were $\pi^{+}$, $\pi^{-}$, $K^{+}$, $K^{-}$, and $\bar{p}$. We extracted the kinetic freeze-out temperature, transverse flow velocity, and kinetic freeze-out volume from the transverse momentum spectra of the particles. We observed that the kinetic freeze-out temperature is rapidity and energy dependent, while the transverse flow velocity does not depend on them. Furthermore, we observed that the kinetic freeze-out volume is energy dependent, but it remains constant with changing the rapidity. We also observed that all three parameters are mass dependent. In addition, with the increase of mass, the kinetic freeze-out temperature increases, and the transverse flow velocity, as well as kinetic freeze-out volume decrease.

## 1. Introduction

To study the dynamics of high-energy collisions, the transverse momentum spectra of the particles are the most important tool to use. The proton–proton (pp) interactions are used as the baseline and are significant for understanding the particle production mechanism [1].

In order to understand the nuclear reaction mechanism and evolution characteristic, the excitation functions of some physical quantities (i.e., different temperatures including initial temperature ($T_{i}$), effective temperature (*T*), chemical freeze-out temperature ($T_{ch}$), and kinetic freeze-out temperature ($T_{0}$) and mean transverse momentum $<p_{T}>$) are very important. We can learn more about the high-energy collision process by analyzing the excitation functions of $T_{0}$, $<p_{T}>$, $T_{i}$, transverse flow velocity ($\beta_{T}$), and kinetic freeze-out volume (*V*), in which the excitation functions of the above parameters can be extracted from the transverse momentum spectra ($p_{T}$) of the particles. The parameters mentioned above were discussed in detail in [2,3,4,5,6,7]. However, we would like to further discuss the temperature here, because temperature is one of the most significant concepts in thermodynamics and statistical mechanics [8], and it describes the excitation degree of the interacting system in subatomic physics.

There are different kinds of temperature discussed in the literature [3,4,5,6,7], which correspond to different collision stages, and it is expected that they vary at different stages due to the system evolution. In the present work, we study the final state particles; therefore, we are interested in the kinetic freeze-out temperature, which occurs at the stage of kinetic freeze-out. In the literature, there are various kinetic freeze-out scenarios including single, double, triple, and multiple kinetic freeze-out scenarios [9,10,11,12,13,14]. In addition, there are various claims and dependencies of the kinetic freeze-out temperature on centrality [5,6,7,8,9,10,13,15,16,17] and collision energy [2,3,10,18]. In our recent work, it was observed that the kinetic freeze-out temperature also depends on coalescence and isospin symmetry [19]. We wonder if $T_{0}$ is also expected to depend on rapidity. For this purpose, we analyzed the final-state-identified particles in different rapidity slices at different energies in pp collisions and extracted the kinetic freeze-out temperature, transverse flow velocity ($\beta_{T}$), and kinetic freeze-out volume from the transverse momentum spectra of the particles by using the blast wave model with the Boltzmann–Gibbs statistics [20,21].

The structure of $\beta_{T}$ and *V* is very complex, and they have been studied in various publications. For centrality dependence, most of the studies agree that $\beta_{T}$ and *V* decrease with decreasing centrality [4,6,7,13,18], but the dependence of $\beta_{T}$ on collision energy is a matter of contradiction [10,17,18].

The remainder of the paper includes the formalism and method, results and discussions, and conclusions.

## 2. Formalism and Method

It is experimentally established that the system that is produced during high-energy collisions has an azimuthal anisotropy due to the difference in flow velocities along various directions. This azimuthal anisotropy occurs due to some initial state geometric effects that rise during the collision process. Therefore, it can be testified that the departing particles must carry some impressions of such effects that can have an impact on the nature of the spectra. In order to include such effects, various models have been suggested [22,23,24,25,26,27,28,29,30,31,32]. In the present work, we used the blast wave model with the Boltzmann–Gibbs statistics (BGBW) [20,21]. The blast wave model is a hydrodynamical-based model. It includes the random thermal motion, as well as the flow properties of the particles. Including the azimuthal anisotropy, the blast wave model gives a complete picture of the quark–gluon plasma (QGP) evolution dynamics. The transverse momentum spectrum of BGBW is given as:

(1) $$\begin{array}{cc} f(p_{T})= & \frac{1}{N}\frac{dN}{dp_{T}}=\frac{1}{N}\frac{gV}{{\left(2\pi \right)}^{2}}p_{T}m_{T}\int_{0}^{R}rdr\\ \; & \times I_{0}\left[\frac{p_{T}\sinh\left(\rho \right)}{T}\right]K_{1}\left[\frac{m_{T}\cosh\left(\rho \right)}{T}\right], \end{array}$$

where *N* represents the number of particles, *g* is the spin degeneracy factor of the particle, which is different for different particles (based on $g_{n}$ = 2$S_{n}$ + 1, while $S_{n}$ is the spin of the particle), *V* denotes the volume of the system under consideration, $m_{T}$ ($m_{T}=\sqrt{p_{T}^{2}+m_{0}^{2}}$) is the transverse mass, $I_{0}$ and $K_{1}$ are the modified Bessel functions, $\rho =\tanh^{-1}\left[\beta \left(r\right)\right]$, and $\beta \left(r\right)=\beta_{S}{\left(r/R\right)}^{n_{0}}$ is the transverse radial flow of the thermal source at radius 0≤r≤*R* with surface velocity $\beta_{S}$. We used $n_{0}$ = 2 to be compatible with [20] in the present work, which closely resembles the hydrodynamic profile as mentioned in [20] and results in $\beta_{T}$ = 0.5β(S). Because the maximum value of β(S) is 1c, the maximum value of $\beta_{T}$ is 0.5c. If $n_{0}$ = 1, it results in $\beta_{T}$ = (2/3)β(S). Therefore, the maximum $\beta_{T}$ is (2/3)c. If $n_{0}$ is used to be a noninteger from that less than one to above two, then it corresponds to the centrality from the center to the periphery. This can lead to a large fluctuation in $\beta_{T}$. The value of $n_{0}$ = 1 or 2 does not effect the result because it is not very sensitive, but if it is taken as a free parameter, then it leads to a large fluctuation in $\beta_{T}$, and naturally, a small change in $T_{0}$ occurs. In general, $\beta_{T}=\left(2/R^{2}\right)\int_{0}^{R}r\beta \left(r\right)dr=2\beta_{S}/\left(n_{0}+2\right)=2\beta_{S}/3$.

We can use Equation (1) for the fitting of $p_{T}$ spectra and extract the parameters $T_{0}$, $\beta_{T}$, and *V*. Equation (1) is applicable in only the soft $p_{T}$ ($p_{T}$ = 2–3 GeV/c) regime of the $p_{T}$ spectra and is valid for a narrow $p_{T}$ range. In the range of $p_{T}$>3 GeV/c, a hard scattering process should be brought into consideration, which was studied in our previous studies [5,8,9,13,16,18].

## 3. Results and Discussions

**Figure 1:**
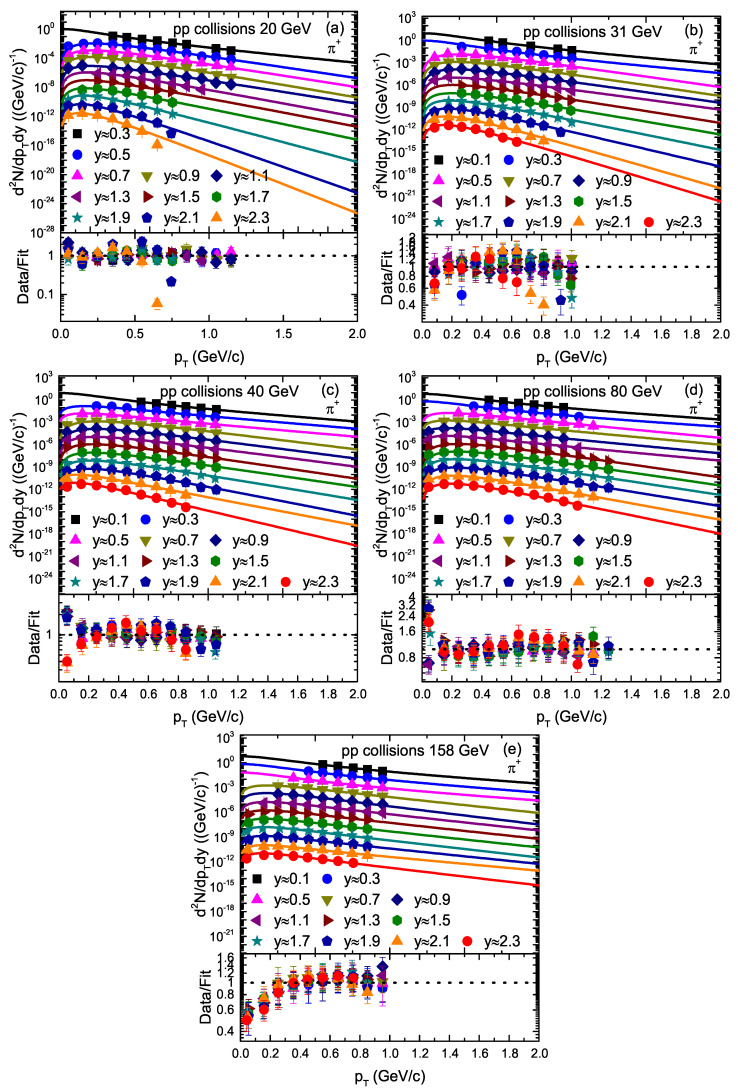
Transverse momentum spectra of $\pi^{+}$ produced in different rapidity slices in pp collisions. Panels (**a**–**e**) correspond to 20 GeV, 31 GeV, 40 GeV, 80 GeV, and 158 GeV energy, respectively. The symbols represent the experimental data of the NA61/SHINE Collaboration measured at CERN [33]. The curves are the results of our fits by the blast wave model with the Boltzmann–Gibbs statistics. The corresponding data/fit ratios are followed in each panel.

**Figure 2:**
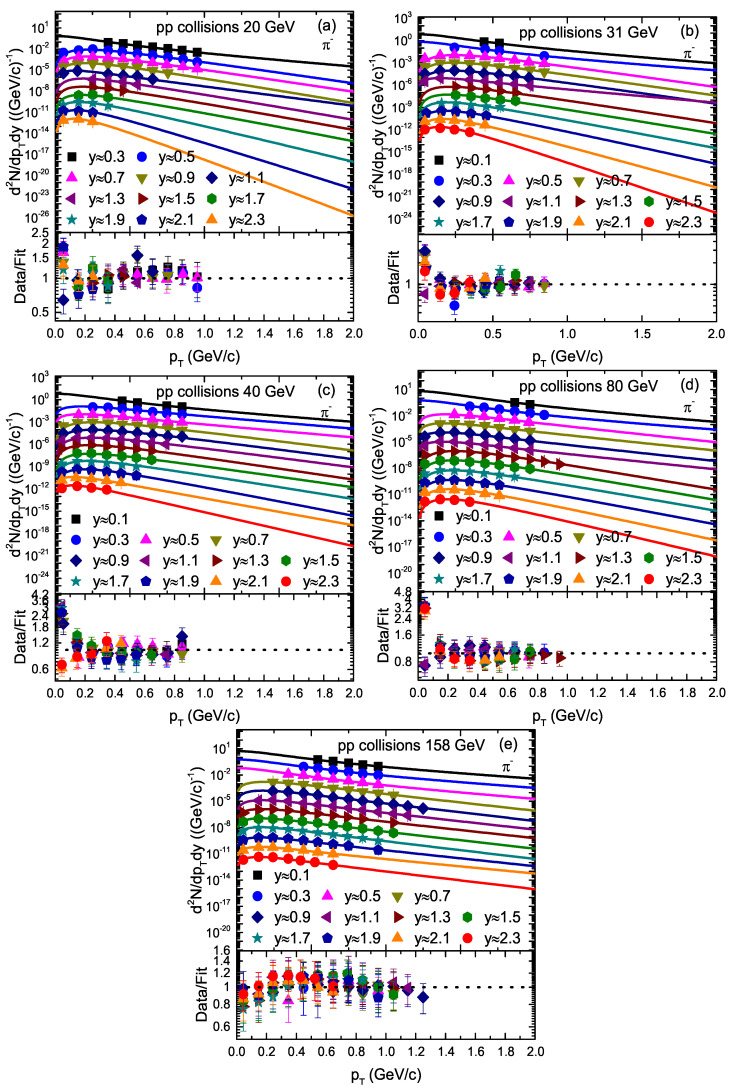
Transverse momentum spectra of $\pi^{-}$ produced in different rapidity slices in pp collisions. Panels (**a**–**e**) correspond to 20 GeV, 31 GeV, 40 GeV, 80 GeV, and 158 GeV energy, respectively. The symbols represent the experimental data of the NA61/SHINE Collaboration measured at CERN [33]. The curves are the results of our fits by the blast wave model with the Boltzmann–Gibbs statistics. The corresponding data/fit ratios are followed in each panel.

The transverse momentum ($p_{T}$) spectra of $\pi^{+}$ and $\pi^{-}$ produced in inelastic (INEL) proton–proton (pp) collisions [33] at different rapidity slices at different energies are represented in Figure 1 and Figure 2, respectively. The symbols represent the experimental data of the NA61/SHINE Collaboration at CERN (European Council for Nuclear Research), and different symbols represent the $p_{T}$ spectra of the particles at different rapidity slices. The collision energy and rapidity slices are labeled in each panel. The solid curves on the experimental data are our fitting by using Equation (1). Different panels correspond to different collision energies.

The lower layer in each panel represents the corresponding ratio of the data/fit. The related values of the free parameters and $\chi^{2}$ and the degrees of freedom (dofs) are presented in Table 1. One can see that Equation (1) provides an approximately good fitting of the data at all rapidity slices.

**Figure 3:**
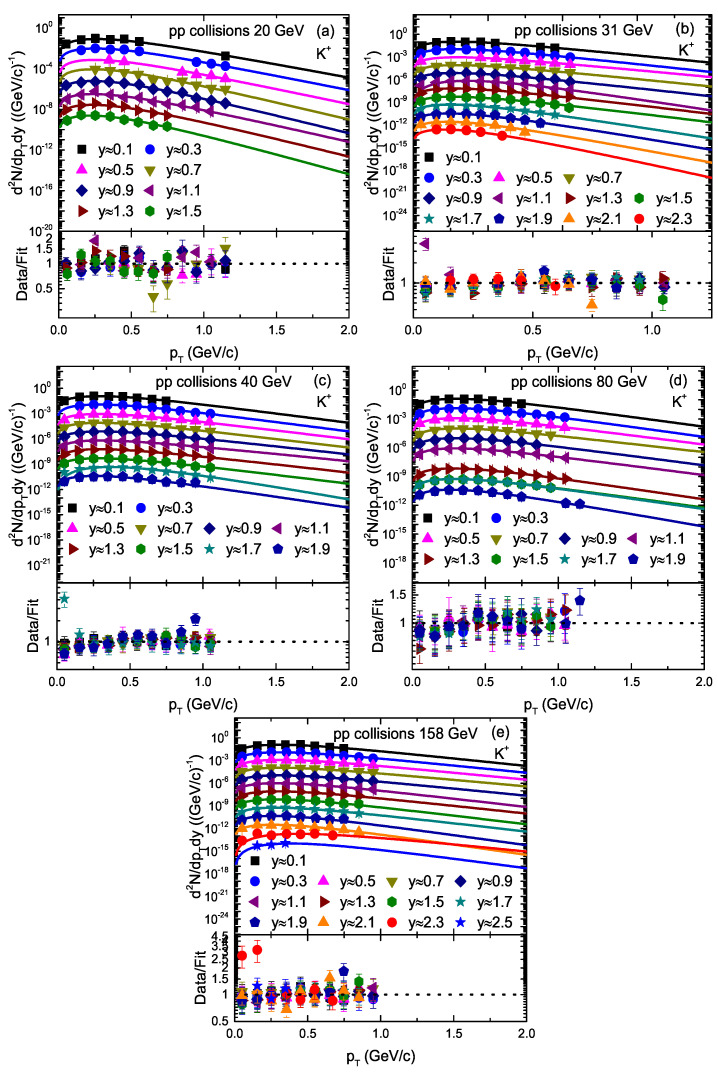
Transverse momentum spectra of $K^{+}$ produced in different rapidity slices in pp collisions. Panels (**a**–**e**) correspond to 20 GeV, 31 GeV, 40 GeV, 80 GeV, and 158 GeV energy, respectively. The symbols represent the experimental data of the NA61/SHINE Collaboration measured at CERN [33]. The curves are the results of our fits by the blast wave model with the Boltzmann–Gibbs statistics. The corresponding data/fit ratios are followed in each panel.

**Figure 4:**
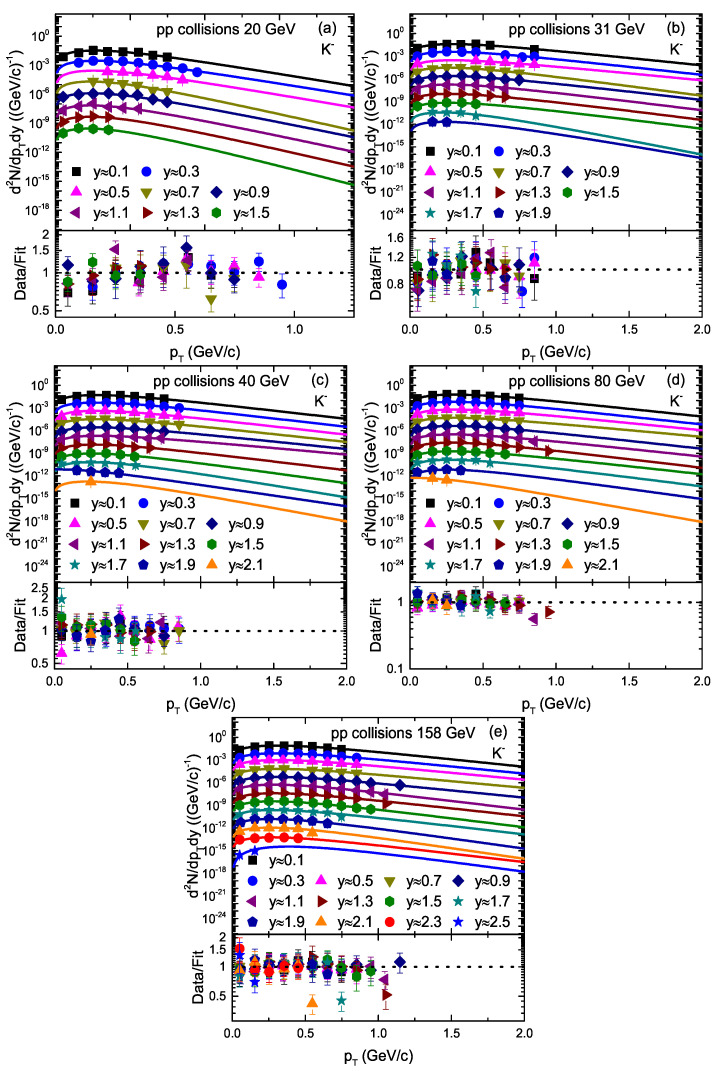
Transverse momentum spectra of $K^{-}$ produced in different rapidity slices in pp collisions. Panels (**a**–**e**) correspond to 20 GeV, 31 GeV, 40 GeV, 80 GeV, and 158 GeV energy, respectively. The symbols represent the experimental data of the NA61/SHINE Collaboration measured at CERN [33]. The curves are the results of our fits by the blast wave model with the Boltzmann–Gibbs statistics. The corresponding data/fit ratios are followed in each panel.

Figure 3 and Figure 4 are similar to Figure 1 and Figure 2, but they show the transverse momentum spectra of $K^{+}$ and $K^{-}$, respectively, at different rapidity slices in pp collisions at different energies. The symbols represent the experimental data of the NA61/SHINE Collaboration [33] measured at CERN. The collision energy and rapidity slices are presented in legends in each panel. The spectra of $K^{-}$ at the 0.5 and 0.7 rapidity slices are scaled by a factor of 1/8 to avoid overlapping of the experimental data and the solid curve with the others. The solid curve over the experimental data shows our fitting result by the blast wave model with the Boltzmann–Gibbs statistics. It can be seen that the Blast wave model provides an approximately good fitting of the data at all rapidity slices.

**Figure 5:**
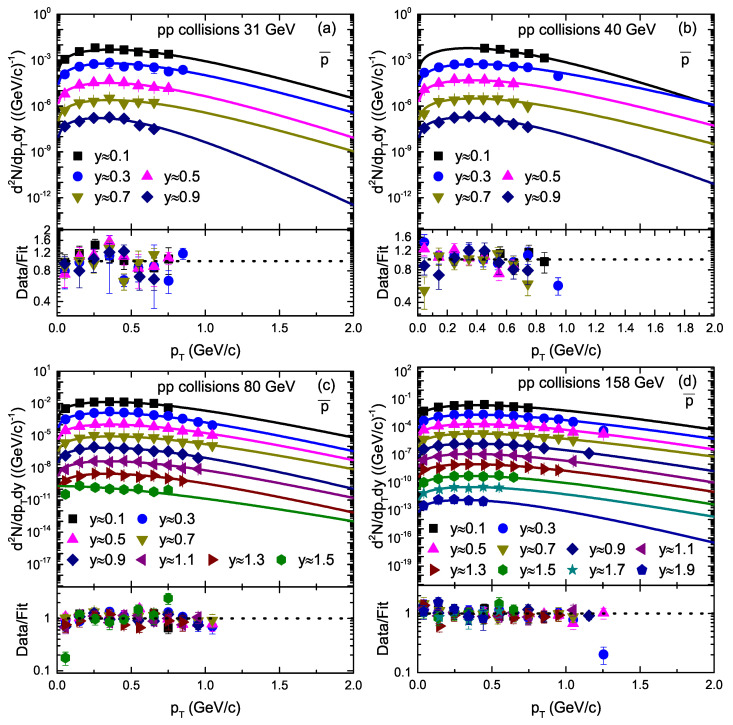
Transverse momentum spectra of $\bar{p}$ produced in different rapidity slices in pp collisions. Panels (**a**–**d**) correspond to 20 GeV, 31 GeV, 40 GeV, 80 GeV, and 158 GeV energy, respectively. The symbols represent the experimental data of the NA61/SHINE Collaboration measured at CERN [33]. The curves are the results of our fits by the blast wave model with the Boltzmann–Gibbs statistics. The corresponding data/fit ratios are followed in each panel.

Figure 5 is similar to Figure 4, but it shows the $p_{T}$ spectra of $\bar{p}$ in pp collisions. The symbols denote the experimental data of the NA61/SHINE Collaboration measured at CERN [33]. Different panels in Figure 5 show different collision energies, and different symbols represent different rapidity slices. The solid curves are the results of our fit by Equation (1) with fluctuations.

We used the least squares method in the fit process to obtain the minimum $\chi^{2}$. From Figure 1, Figure 2, Figure 3, Figure 4 and Figure 5, the $\chi^{2}$ is large in some cases, which shows that the dispersion between the curve and data is large; however, the fitting is approximately acceptable, but in most cases, the model results describe the experimental data well in the $p_{T}$ spectra of the particles produced in different rapidity slices. Each panel in each figure is followed by the corresponding result of its data/fit in order to show the dispersion of the curve from the data. In fact, the data/fit ratio is large in some cases due to the large dispersion between the curve and data.

It should be noted that the data used in this work are from a fixed target experiment, where energies are in the lab frame. Therefore, we needed to convert it to the center-of-mass energies. The corresponding 20 GeV, 31 GeV, 40 GeV, 80 GeV, and 158 GeV/c energies in the lab frame are equal to 6.3 GeV, 7.7 GeV, 8.8 GeV, 12.3 GeV, and 17.3 GeV, respectively, in the center-of-mass frame. In addition, The rapidity of the particle was measured in the cms system as *y* = a tanh$\beta_{L}$, where $\beta_{L}$ represents the longitudinal component of the velocity and is given by $\beta_{L}$ = $p_{L}$/E with c = 1, whereas E and $p_{L}$ are the energy and longitudinal momentum in the cms frame.

**Figure 6:**
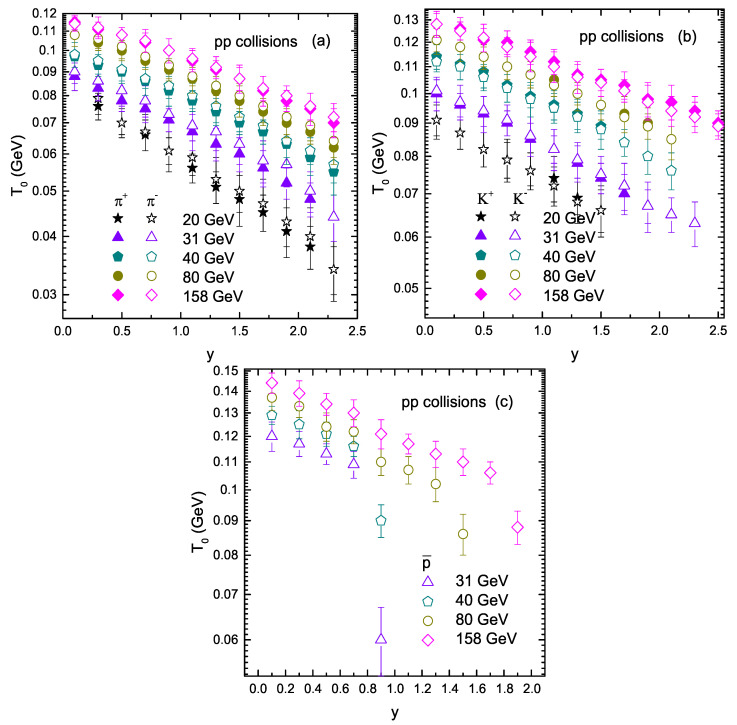
Dependence of $T_{0}$ on rapidity and collision energy.

To study the change in the trend of the parameters with rapidity and collision energy, Figure 6 shows the dependencies of the kinetic freeze-out temperature ($T_{0}$) on rapidity and energy for the production of different particles in pp collisions. Panels (a), (b), and (c) correspond to pion, kaon, and antiproton, respectively. The closed and open symbols in Panels (a)–(c) represent the positively and negatively charged particles, respectively. The trend of the symbols from left to right shows the dependence of the kinetic freeze-out temperature ($T_{0}$) on rapidity, while the dependence of $T_{0}$ on energy is shown by the symbols from top to bottom. One can see that $T_{0}$ increases with the increase of the collision energy. The reason behind this is that when the energy increases, the collision becomes more violent and transfers more energy, which results in higher excitation of the system, and naturally, the system with a high degree of excitation has a high $T_{0}$. On the other hand, the kinetic freeze-out temperature ($T_{0}$) decreases with the increase of rapidity from the midrapidity region to the forward rapidity, because when rapidity increases, the energy transfer in the system decreases due to the large penetration between participant nucleons. In the meantime, due to fewer produced particles taking part in the scattering process, the degree of multiple scattering also decreases, and $p_{T}$ decreases due to both factors. In addition, it was observed that $T_{0}$ increases for heavier particles, which shows the mass differential kinetic freeze-out scenario.

**Figure 7:**
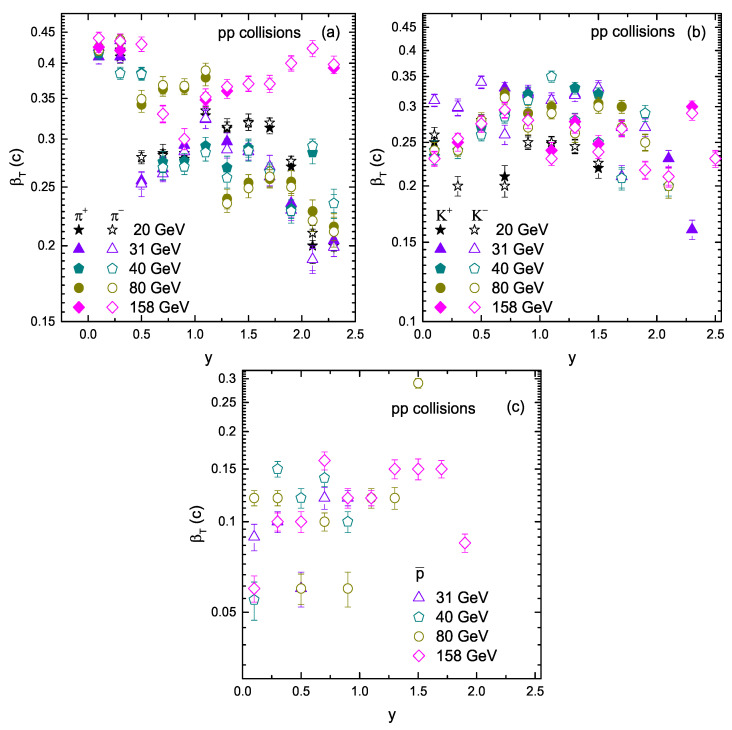
Dependence of $\beta_{T}$ on rapidity and collision energy.

Figure 7 is similar to Figure 6, but it shows the dependence of $\beta_{T}$ on rapidity and energy. At present, we did not observe any dependence of $\beta_{T}$ on rapidity and energy. Although there is an energy dependence of $\beta_{T}$ in the literature [10,17], we can study it in more detail by analyzing more data for different particles in different collisions with different models. Furthermore, $\beta_{T}$ is mass dependent, and it is larger for lighter particles.

We would like to point out that QGP-like properties have been reported at LHC energies in pp collisions, where the values of $T_{0}$ and $\beta_{T}$ are reported to be 163 MeV and 10 MeV and 0.49 and 0.02, respectively [34]. It is also not possible to observe any such effect at the energies under consideration, but the relevant parameter can still be checked for low energies to understand the nature of the collisions in the final state in comparison to high-energy pp collisions. The values observed in our case are small compared to the one listed above, which shows that no such effect is observed in low-energy pp collisions.

Both $T_{0}$ and $\beta_{T}$ show mass dependency, which reflects the formation time dependence. Hydrodynamically, heavier particles come out of the system earlier in time with smaller radial flow velocities. This shows that as the mass increases, the formation time, as well as $\beta_{T}$ decrease, whereas $T_{0}$ increases. Indeed, there are various hydrodynamical simulations that have observed one set of parameters for all the particles (common freeze-out temperature, as well as transverse flow velocity), but their explanation was different. Besides, we can obtain different results from different models; even from the same model, we can obtain different results if the method is different and the limitations and conditions are different. The selection of $T_{0}$ and $\beta_{T}$ is very technical and complex, as in some cases, it depends on the range of the $p_{T}$ spectra, and it may also depend on the value of $n_{0}$. If the value of $n_{0}$ is one or two, it does not have an effect on the results of the parameters, but if it is taken as a free parameter, then the free parameters (especially $\beta_{T}$) fluctuate obviously.

**Figure 8:**
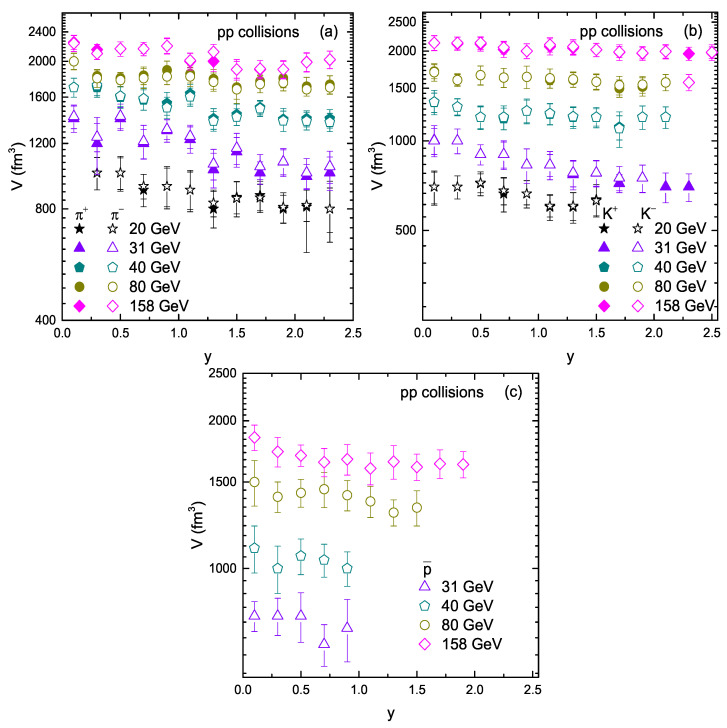
Dependence of *V* on rapidity and collision energy.

Figure 8 is similar to Figure 6 and Figure 7, but it shows the dependence of *V* on rapidity and energy. One can see that the kinetic freeze-out volume increases with energy. The reason behind this is that larger initial bulk system exists at high energy. The evolution time becomes longer at higher energies, and it corresponds to a larger partonic system; naturally, the kinetic freeze-out volume becomes larger in a large partonic system. In the present work, we did not observe any clear dependence of *V* on rapidity because the trend of *V* is almost constant. However, we may consider more analysis for the detailed study of the dependence of *V* on rapidity in the future. Additionally, *V* is observed to be mass dependent. The larger the mass of the particle, the smaller *V* is, which shows the early freeze-out of massive particles. This result is consistent with our previous results [4,6,13].

## 4. Summary and Conclusions

We summarize here our main observations and conclusions.

(a) The transverse momentum spectra of positively and negatively charged particles produced in inelastic proton–proton collisions in different rapidity slices were studied by the blast wave model with the Boltzmann–Gibbs statistics. The results were in good agreement with the experimental data measured by the NA61/SHINE Collaboration at CERN over an energy range from 20 GeV to 158 GeV.

(b) The kinetic freeze-out temperature increased with the increase of the collision energy due to the large deposition of the energy in the system at higher energies, and it decreased with the increase of rapidity because of the lesser energy transfer in the system due to the large penetration between participating nucleons. It was also observed that the kinetic freeze-out temperature increased with mass.

(c) The transverse flow velocity was observed to have no dependence on rapidity and energy. However it was dependent on the mass of the particle. Massive particles have smaller $\beta_{T}$.

(d) There was no dependence of the kinetic freeze-out volume observed on rapidity in the present work. However, *V* increased with the increase of the energy due to the longer evolution time at higher energies. The volume differential scenario was also observed as the massive particles have smaller *V* and they froze out early.

## Figures and Tables

**Table 1:** List of the parameters (– is used in some places instead of the dofs. In fact, it is not the fit result. If dofs < 0, then we put - instead of the negative values.).

Collisions	Rapidity	Particle	$T_{0}$ (GeV)	$\beta_{T}$ (c)	$V({\mathrm{fm}}^{3})$	$N_{0}$	$\chi^{2}$/dofs
Figure 1	y = 0.3	$\pi^{+}$	0.076±0.005	0.410±0.010	1000±100	$9.5\times 10^{-7}\pm 5\times 10^{-8}$	5/4
p-p	y = 0.5	–	0.070±0.004	0.280±0.007	1000±108	$5.0\times 10^{-7}\pm 4\times 10^{-8}$	4/8
20 GeV	y = 0.7	–	0.066±0.005	0.284±0.010	900±90	$6\times 10^{-8}\pm 6\times 10^{-9}$	3.5/8
	y = 0.9	–	0.061±0.006	0.284±0.011	920±120	$7\times 10^{-9}\pm 6\times 10^{-10}$	9/8
	y = 1.1	–	0.056±0.004	0.328±0.008	900±105	$4.5\times 10^{-10}\pm 4\times 10^{-11}$	11/7
	y = 1.3	–	0.051±0.004	0.312±0.012	800±90	$6.4\times 10^{-11}\pm 5\times 10^{-12}$	4/4
	y = 1.5	–	0.048±0.006	0.320±0.010	860±85	$4.9\times 10^{-12}\pm 5\times 10^{-13}$	1/2
	y = 1.7	–	0.045±0.004	0.313±0.007	870±80	$3.8\times 10^{-13}\pm 4\times 10^{-14}$	9/3
	y = 1.9	–	0.041±0.005	0.270±0.011	800±70	$4\times 10^{-14}\pm 6\times 10^{-15}$	5/4
	y = 2.1	–	0.038±0.004	0.200±0.013	810±200	$2.5\times 10^{-15}\pm 4\times 10^{-16}$	118/4
	y = 2.3	–	0.034±0.005	0.200±0.008	800±150	$1.5\times 10^{-16}\pm 7\times 10^{-17}$	120/3
Figure 2	y = 0.3	$\pi^{-}$	0.079±0.006	0.421±0.013	1000±100	$6.5\times 10^{-7}\pm 6\times 10^{-8}$	3/3
p-p	y = 0.5	–	0.070±0.005	0.280±0.008	1000±98	$3.5\times 10^{-7}\pm 5\times 10^{-8}$	6/5
20 GeV	y = 0.7	–	0.067±0.006	0.282±0.011	920±70	$4\times 10^{-8}\pm 4\times 10^{-9}$	3/6
	y = 0.9	–	0.061±0.004	0.278±0.009	920±110	$4.5\times 10^{-9}\pm 6\times 10^{-10}$	7/4
	y = 1.1	–	0.059±0.005	0.334±0.011	900±115	$7.2\times 10^{-11}\pm 7\times 10^{-12}$	8/2
	y = 1.3	–	0.053±0.006	0.323±0.010	830±69	$3.6\times 10^{-11}\pm 4\times 10^{-12}$	3/0
	y = 1.5	–	0.050±0.005	0.319±0.012	854±93	$2.2\times 10^{-12}\pm 6\times 10^{-13}$	0.2/-
	y = 1.7	–	0.047±0.006	0.319±0.009	857±75	$1.5\times 10^{-13}\pm 5\times 10^{-14}$	1/-
	y = 1.9	–	0.043±0.005	0.276±0.010	808±75	$1.5\times 10^{-14}\pm 5\times 10^{-15}$	1/-
	y = 2.1	–	0.040±0.006	0.210±0.011	819±80	$1\times 10^{-15}\pm 5\times 10^{-16}$	10/-
	y = 2.3	–	0.034±0.004	0.200±0.008	800±110	$6\times 10^{-17}\pm 7\times 10^{-18}$	1/-
Figure 1	y = 0.1	$\pi^{+}$	0.088±0.006	0.410±0.011	1400±114	$7\times 10^{-6}\pm 4\times 10^{-7}$	0.1/1
p-p	y = 0.3	–	0.083±0.005	0.410±0.009	1200±210	$7.9\times 10^{-7}\pm 5\times 10^{-8}$	21/4
31 GeV	y = 0.5	–	0.078±0.004	0.256±0.008	1400±100	$4.2\times 10^{-7}\pm 3\times 10^{-8}$	1.5/7
	y = 0.7	–	0.075±0.005	0.268±0.012	1200±108	$4.8\times 10^{-8}\pm 4\times 10^{-9}$	2/7
	y = 0.9	–	0.071±0.004	0.293±0.009	1300±90	$4.8\times 10^{-9}\pm 4\times 10^{-10}$	2/7
	y = 1.1	–	0.067±0.006	0.303±0.011	1230±100	$4.2\times 10^{-10}\pm 5\times 10^{-11}$	2/6
	y = 1.3	–	0.063±0.005	0.297±0.010	1020±110	$4.5\times 10^{-11}\pm 4\times 10^{-12}$	3.5/5
	y = 1.5	–	0.060±0.005	0.279±0.008	1140±105	$3.1\times 10^{-12}\pm 4\times 10^{-13}$	8/5
	y = 1.7	–	0.056±0.005	0.260±0.009	1000±70	$3.6\times 10^{-13}\pm 4\times 10^{-14}$	31/6
	y = 1.9	–	0.052±0.004	0.233±0.012	1070±83	$2.7\times 10^{-14}\pm 4\times 10^{-15}$	25/6
	y = 2.1	–	0.048±0.004	0.190±0.010	978±76	$2.6\times 10^{-15}\pm 5\times 10^{-16}$	80/5
	y = 2.3	–	0.044±0.005	0.204±0.007	1000±104	$1.8\times 10^{-16}\pm 4\times 10^{-17}$	10/3
Figure 2	y = 0.1	$\pi^{-}$	0.090±0.005	0.425±0.010	1420±104	$5.5\times 10^{-6}\pm 6\times 10^{-7}$	0.2/-
p-p	y = 0.3	–	0.086±0.006	0.436±0.011	1250±110	$5.9\times 10^{-7}\pm 4\times 10^{-8}$	16/-
31 GeV	y = 0.5	–	0.082±0.006	0.253±0.012	1423±109	$3.4\times 10^{-7}\pm 5\times 10^{-8}$	8/5
	y = 0.7	–	0.078±0.006	0.263±0.008	1216±128	$3.8\times 10^{-8}\pm 7\times 10^{-9}$	11/5
	y = 0.9	–	0.073±0.006	0.286±0.012	1310±80	$3\times 10^{-9}\pm 5\times 10^{-10}$	10/4
	y = 1.1	–	0.069±0.005	0.324±0.012	1255±90	$7\times 10^{-11}\pm 7\times 10^{-12}$	2/3
	y = 1.3	–	0.067±0.006	0.288±0.009	1057±100	$2.5\times 10^{-11}\pm 5\times 10^{-12}$	0.3/1
	y = 1.5	–	0.063±0.006	0.286±0.009	1166±112	$2\times 10^{-12}\pm 6\times 10^{-13}$	3.5/1
	y = 1.7	–	0.058±0.006	0.270±0.012	1040±80	$2\times 10^{-13}\pm 4\times 10^{-14}$	6/1
	y = 1.9	–	0.057±0.006	0.229±0.009	1070±92	$1.4\times 10^{-14}\pm 4\times 10^{-15}$	6/1
	y = 2.1	–	0.050±0.005	0.190±0.008	1000±93	$1.2\times 10^{-15}\pm 4\times 10^{-16}$	10/1
	y = 2.3	–	0.044±0.005	0.199±0.007	1040±104	$9.4\times 10^{-17}\pm 4\times 10^{-18}$	9/-
Figure 1	y = 0.1	$\pi^{+}$	0.097±0.005	0.415±0.010	1700±100	$6.7\times 10^{-6}\pm 5\times 10^{-7}$	0.1/2
p-p	y = 0.3	–	0.093±0.005	0.385±0.007	1700±103	$1\times 10^{-7}\pm 4\times 10^{-8}$	3/5
40 GeV	y = 0.5	–	0.090±0.005	0.385±0.009	1600±106	$1.1\times 10^{-7}\pm 4\times 10^{-8}$	3.5/6
	y = 0.7	–	0.086±0.006	0.275±0.011	1575±100	$3.8\times 10^{-8}\pm 5\times 10^{-9}$	14/7
	y = 0.9	–	0.082±0.006	0.276±0.008	1540±110	$3.7\times 10^{-9}\pm 4\times 10^{-10}$	10/7
	y = 1.1	–	0.078±0.004	0.292±0.010	1633±100	$3.4\times 10^{-10}\pm 4\times 10^{-11}$	6/7
	y = 1.3	–	0.074±0.005	0.269±0.012	1400±90	$3.5\times 10^{-11}\pm 4\times 10^{-12}$	4/7
	y = 1.5	–	0.070±0.004	0.290±0.010	1437±96	$2.6\times 10^{-12}\pm 4\times 10^{-13}$	3/6
	y = 1.7	–	0.067±0.004	0.265±0.007	1500±67	$2.1\times 10^{-13}\pm 5\times 10^{-14}$	21/7
	y = 1.9	–	0.063±0.006	0.230±0.008	1394±100	$1.8\times 10^{-14}\pm 4\times 10^{-15}$	20/7
	y = 2.1	–	0.059±0.005	0.285±0.012	1400±70	$4.5\times 10^{-15}\pm 5\times 10^{-16}$	58/5
	y = 2.3	–	0.055±0.006	0.235±0.008	1400±80	$3.2\times 10^{-16}\pm 5\times 10^{-17}$	41/5
Figure 2	y = 0.1	$\pi^{-}$	0.098±0.006	0.418±0.010	1700±100	$4.6\times 10^{-6}\pm 4\times 10^{-7}$	0.6/-
p-p	y = 0.3	–	0.095±0.006	0.385±0.009	1720±105	$8\times 10^{-7}\pm 6\times 10^{-8}$	1/3
40 GeV	y = 0.5	–	0.091±0.005	0.384±0.009	1611±100	$7.6\times 10^{-8}\pm 7\times 10^{-9}$	2/4
	y = 0.7	–	0.087±0.004	0.269±0.012	1584±110	$2.5\times 10^{-8}\pm 6\times 10^{-9}$	7/5
	y = 0.9	–	0.084±0.005	0.270±0.008	1500±100	$2.6\times 10^{-9}\pm 5\times 10^{-10}$	7/5
	y = 1.1	–	0.080±0.005	0.285±0.009	1603±90	$2.1\times 10^{-10}\pm 6\times 10^{-11}$	14/4
	y = 1.3	–	0.076±0.006	0.259±0.010	1380±85	$2.4\times 10^{-11}\pm 5\times 10^{-12}$	10/3
	y = 1.5	–	0.072±0.005	0.287±0.011	1415±73	$1.6\times 10^{-12}\pm 6\times 10^{-13}$	4/2
	y = 1.7	–	0.069±0.006	0.265±0.009	1490±71	$1.6\times 10^{-13}\pm 5\times 10^{-14}$	17/2
	y = 1.9	–	0.064±0.005	0.228±0.010	1381±87	$1.3\times 10^{-14}\pm 7\times 10^{-15}$	14/2
	y = 2.1	–	0.061±0.004	0.292±0.008	1380±76	$2\times 10^{-16}\pm 5\times 10^{-17}$	12/1
	y = 2.3	–	0.057±0.005	0.235±0.013	1370±78	$1.3\times 10^{-17}\pm 5\times 10^{-18}$	73/-
Figure 1	y = 0.1	$\pi^{+}$	0.108±0.006	0.425±0.011	2000±100	$4.9\times 10^{-6}\pm 4\times 10^{-7}$	0.2/2
p-p	y = 0.3	–	0.104±0.004	0.435±0.008	1824±70	$5.2\times 10^{-7}\pm 5\times 10^{-8}$	1/4
80 GeV	y = 0.5	–	0.100±0.005	0.342±0.010	1800±66	$1.1\times 10^{-7}\pm 3\times 10^{-8}$	1/6
	y = 0.7	–	0.095±0.004	0.363±0.010	1829±100	$1.1\times 10^{-8}\pm 7\times 10^{-9}$	0.4/5
	y = 0.9	–	0.091±0.005	0.364±0.009	1892±110	$9.5\times 10^{-10}\pm 6\times 10^{-11}$	5/6
	y = 1.1	–	0.087±0.006	0.379±0.012	1850±103	$8.8\times 10^{-11}\pm 7\times 10^{-12}$	7/7
	y = 1.3	–	0.082±0.005	0.239±0.009	1790±108	$3\times 10^{-11}\pm 6\times 10^{-12}$	14/9
	y = 1.5	–	0.078±0.006	0.259±0.008	1700±160	$3\times 10^{-12}\pm 7\times 10^{-13}$	11/9
	y = 1.7	–	0.074±0.005	0.258±0.009	1766±70	$2.7\times 10^{-13}\pm 6\times 10^{-14}$	6/9
	y = 1.9	–	0.070±0.005	0.255±0.011	1800±103	$2\times 10^{-14}\pm 5\times 10^{-15}$	15/9
	y = 2.1	–	0.067±0.004	0.228±0.010	1710±100	$1.7\times 10^{-15}\pm 5\times 10^{-15}$	13/8
	y = 2.3	–	0.062±0.005	0.215±0.011	1730±97	$1.4\times 10^{-16}\pm 7\times 10^{-17}$	17/7
Figure 2	y = 0.1	$\pi^{-}$	0.108±0.006	0.437±0.008	2000±104	$4.4\times 10^{-6}\pm 6\times 10^{-7}$	1/-
p-p	y = 0.3	–	0.106±0.005	0.457±0.009	1800±100	$4.5\times 10^{-7}\pm 5\times 10^{-8}$	1/2
80 GeV	y = 0.5	–	0.102±0.006	0.349±0.012	1780±72	$9\times 10^{-8}\pm 5\times 10^{-9}$	1/2
	y = 0.7	–	0.097±0.006	0.368±0.012	1800±110	$8.5\times 10^{-9}\pm 7\times 10^{-10}$	1/3
	y = 0.9	–	0.093±0.006	0.367±0.010	1820±90	$7.3\times 10^{-10}\pm 4\times 10^{-11}$	4/4
	y = 1.1	–	0.088±0.004	0.389±0.011	1820±108	$6.3\times 10^{-11}\pm 6\times 10^{-12}$	4/4
	y = 1.3	–	0.084±0.004	0.235±0.008	1760±100	$2.2\times 10^{-11}\pm 6\times 10^{-12}$	17/6
	y = 1.5	–	0.080±0.005	0.249±0.009	1680±100	$2\times 10^{-12}\pm 5\times 10^{-13}$	16/4
	y = 1.7	–	0.076±0.006	0.260±0.010	1735±90	$1.4\times 10^{-13}\pm 4\times 10^{-14}$	16/3
	y = 1.9	–	0.072±0.006	0.250±0.008	1750±86	$1.1\times 10^{-14}\pm 6\times 10^{-15}$	9/2
	y = 2.1	–	0.069±0.005	0.220±0.009	1680±80	$1\times 10^{-15}\pm 5\times 10^{-15}$	14/2
	y = 2.3	–	0.064±0.005	0.211±0.012	1700±70	$7\times 10^{-17}\pm 5\times 10^{-18}$	15/-
Figure 1	y = 0.1	$\pi^{+}$	0.115±0.004	0.425±0.008	2250±100	$4.2\times 10^{-6}\pm 5\times 10^{-7}$	0.3/1
p-p	y = 0.3	–	0.111±0.004	0.420±0.009	2150±70	$4.7\times 10^{-7}\pm 6\times 10^{-8}$	0.4/2
158 GeV	y = 0.5	–	0.108±0.004	0.430±0.012	2160±96	$4.4\times 10^{-8}\pm 5\times 10^{-9}$	1/3
	y = 0.7	–	0.104±0.005	0.330±0.011	2160±60	$9.2\times 10^{-9}\pm 4\times 10^{-10}$	0.5/4
	y = 0.9	–	0.100±0.006	0.300±0.012	2200±110	$1\times 10^{-9}\pm 6\times 10^{-10}$	4/4
	y = 1.1	–	0.095±0.005	0.348±0.010	2000±106	$9.5\times 10^{-11}\pm 7\times 10^{-12}$	5/5
	y = 1.3	–	0.091±0.004	0.360±0.010	2000±122	$9\times 10^{-12}\pm 6\times 10^{-13}$	16/5
	y = 1.5	–	0.087±0.005	0.370±0.011	1900±120	$9\times 10^{-13}\pm 7\times 10^{-14}$	13/5
	y = 1.7	–	0.082±0.006	0.370±0.008	1900±108	$8.6\times 10^{-14}\pm 6\times 10^{-15}$	21/5
	y = 1.9	–	0.078±0.004	0.280±0.012	1900±107	$7.3\times 10^{-15}\pm 5\times 10^{-16}$	19/5
	y = 2.1	–	0.075±0.006	0.423±0.007	1990±120	$5.5\times 10^{-16}\pm 5\times 10^{-17}$	26/5
	y = 2.3	–	0.070±0.005	0.393±0.008	2019±110	$5\times 10^{-17}\pm 7\times 10^{-18}$	42/4
Figure 2	y = 0.1	$\pi^{-}$	0.114±0.004	0.440±0.010	2235±110	$3.9\times 10^{-6}\pm 7\times 10^{-7}$	0.4/1
p-p	y = 0.3	–	0.112±0.006	0.435±0.012	2100±80	$4.4\times 10^{-7}\pm 7\times 10^{-8}$	0.1/2
158 GeV	y = 0.5	–	0.108±0.004	0.430±0.012	2160±96	$4\times 10^{-8}\pm 5\times 10^{-9}$	1.5/3
	y = 0.7	–	0.105±0.006	0.330±0.010	2160±90	$8.4\times 10^{-9}\pm 4\times 10^{-10}$	1/4
	y = 0.9	–	0.100±0.006	0.330±0.012	2200±100	$8\times 10^{-10}\pm 5\times 10^{-11}$	1/7
	y = 1.1	–	0.096±0.005	0.352±0.011	2010±91	$7\times 10^{-11}\pm 4\times 10^{-12}$	2/7
	y = 1.3	–	0.092±0.005	0.366±0.010	2120±102	$6.4\times 10^{-12}\pm 4\times 10^{-13}$	3/6
	y = 1.5	–	0.087±0.006	0.370±0.010	1900±100	$6\times 10^{-13}\pm 5\times 10^{-15}$	3.5/7
	y = 1.7	–	0.083±0.005	0.370±0.012	1909±98	$5.6\times 10^{-14}\pm 5\times 10^{-15}$	5/6
	y = 1.9	–	0.080±0.004	0.400±0.010	1900±100	$4\times 10^{-15}\pm 7\times 10^{-16}$	2/5
	y = 2.1	–	0.076±0.005	0.423±0.013	1988±110	$3.2\times 10^{-16}\pm 5\times 10^{-17}$	1/3
	y = 2.3	–	0.072±0.005	0.398±0.013	2020±110	$2\times 10^{-17}\pm 5\times 10^{-18}$	1/3
Figure 3	y = 0.1	$K^{+}$	0.091±0.005	0.250±0.010	700±85	$1.5\times 10^{-6}\pm 5\times 10^{-7}$	6/3
p-p	y = 0.3	–	0.087±0.005	0.200±0.010	700±62	$1.7\times 10^{-7}\pm 4\times 10^{-8}$	5/5
20 GeV	y = 0.5	–	0.082±0.005	0.280±0.008	720±66	$1.2\times 10^{-8}\pm 6\times 10^{-9}$	7/2
	y = 0.7	–	0.079±0.006	0.210±0.012	665±88	$1.45\times 10^{-9}\pm 5\times 10^{-10}$	27/6
	y = 0.9	–	0.076±0.005	0.250±0.009	665±70	$1\times 10^{-10}\pm 6\times 10^{-11}$	5/8
	y = 1.1	–	0.074±0.006	0.248±0.009	600±60	$6\times 10^{-12}\pm 6\times 10^{-13}$	15/7
	y = 1.3	–	0.069±0.004	0.245±0.009	600±71	$5\times 10^{-13}\pm 7\times 10^{-14}$	8/4
	y = 1.5	–	0.066±0.006	0.219±0.011	635±80	$3.9\times 10^{-14}\pm 7\times 10^{-15}$	12/4
Figure 4	y = 0.1	$K^{-}$	0.091±0.006	0.260±0.010	700±92	$6.1\times 10^{-7}\pm 4\times 10^{-8}$	10/6
p-p	y = 0.3	–	0.087±0.005	0.200±0.010	700±62	$5.4\times 10^{-8}\pm 5\times 10^{-9}$	6/5
20 GeV	y = 0.5	–	0.082±0.005	0.280±0.008	722±53	$4.8\times 10^{-9}\pm 5\times 10^{-10}$	3/2
	y = 0.7	–	0.079±0.005	0.200±0.011	680±70	$3.3\times 10^{-10}\pm 7\times 10^{-11}$	8/2
	y = 0.9	–	0.076±0.004	0.250±0.007	665±55	$2.2\times 10^{-11}\pm 5\times 10^{-12}$	5/4
	y = 1.1	–	0.072±0.005	0.250±0.008	605±50	$1.3\times 10^{-12}\pm 5\times 10^{-13}$	8/2
	y = 1.3	–	0.068±0.005	0.245±0.008	605±65	$8.5\times 10^{-14}\pm 5\times 10^{-15}$	2/-
	y = 1.5	–	0.064±0.005	0.225±0.009	630±70	$4.6\times 10^{-15}\pm 4\times 10^{-16}$	2/-
Figure 3	y = 0.1	$K^{+}$	0.101±0.006	0.310±0.010	1004±120	$1.6\times 10^{-6}\pm 6\times 10^{-7}$	1.5/5
p-p	y = 0.3	–	0.097±0.006	0.298±0.013	1000±95	$1.6\times 10^{-7}\pm 4\times 10^{-8}$	1/7
31 GeV	y = 0.5	–	0.094±0.006	0.340±0.010	900±70	$1.6\times 10^{-8}\pm 5\times 10^{-9}$	0.2/4
	y = 0.7	–	0.091±0.004	0.330±0.010	900±90	$1.4\times 10^{-9}\pm 6\times 10^{-10}$	4.5/7
	y = 0.9	–	0.086±0.005	0.323±0.012	830±110	$1.3\times 10^{-10}\pm 5\times 10^{-11}$	5/7
	y = 1.1	–	0.082±0.004	0.310±0.008	830±70	$3.2\times 10^{-11}\pm 4\times 10^{-12}$	15/7
	y = 1.3	–	0.079±0.005	0.320±0.012	770±85	$1.2\times 10^{-12}\pm 5\times 10^{-13}$	12/7
	y = 1.5	–	0.075±0.006	0.330±0.009	780±76	$9.6\times 10^{-14}\pm 5\times 10^{-15}$	9/7
	y = 1.7	–	0.072±0.005	0.270±0.011	720±50	$8\times 10^{-15}\pm 7\times 10^{-16}$	9/6
	y = 1.9	–	0.067±0.005	0.270±0.012	750±78	$5\times 10^{-16}\pm 5\times 10^{-17}$	4.5/5
	y = 2.1	–	0.068±0.006	0.230±0.010	700±80	$4\times 10^{-17}\pm 4\times 10^{-18}$	33/4
	y = 2.3	–	0.063±0.004	0.200±0.008	700±75	$3.6\times 10^{-18}\pm 5\times 10^{-19}$	1/1
Figure 4	y = 0.1	$K^{-}$	0.100±0.004	0.310±0.009	1000±100	$6\times 10^{-7}\pm 4\times 10^{-8}$	1.5/3
p-p	y = 0.3	–	0.096±0.006	0.299±0.013	1000±95	$5.5\times 10^{-8}\pm 4\times 10^{-9}$	5/4
31 GeV	y = 0.5	–	0.093±0.005	0.340±0.011	900±70	$5\times 10^{-9}\pm 5\times 10^{-10}$	0.4/2
	y = 0.7	–	0.090±0.006	0.260±0.013	900±100	$4.4\times 10^{-10}\pm 4\times 10^{-11}$	2/4
	y = 0.9	–	0.085±0.006	0.320±0.010	830±110	$4\times 10^{-11}\pm 5\times 10^{-12}$	4.5/4
	y = 1.1	–	0.082±0.006	0.310±0.012	830±90	$2.9\times 10^{-12}\pm 7\times 10^{-13}$	6/3
	y = 1.3	–	0.078±0.005	0.318±0.010	780±80	$1.8\times 10^{-13}\pm 5\times 10^{-14}$	1/3
	y = 1.5	–	0.074±0.005	0.330±0.013	780±80	$1.2\times 10^{-14}\pm 5\times 10^{-15}$	0.4/1
	y = 1.7	–	0.070±0.004	0.210±0.012	750±70	$5\times 10^{-16}\pm 5\times 10^{-17}$	5/-
	y = 1.9	–	0.067±0.006	0.270±0.012	750±80	$3.1\times 10^{-17}\pm 6\times 10^{-18}$	0.2/-
Figure 3	y = 0.1	$K^{+}$	0.114±0.005	0.233±0.010	1345±100	$1.25\times 10^{-6}\pm 5\times 10^{-7}$	6/7
p-p	y = 0.3	–	0.111±0.006	0.240±0.010	1300±80	$1.3\times 10^{-7}\pm 6\times 10^{-8}$	1.5/6
40 GeV	y = 0.5	–	0.108±0.006	0.270±0.012	1200±100	$1.1\times 10^{-8}\pm 4\times 10^{-9}$	9/7
	y = 0.7	–	0.103±0.006	0.290±0.010	1188±100	$1\times 10^{-9}\pm 7\times 10^{-10}$	3/7
	y = 0.9	–	0.099±0.004	0.320±0.012	1260±110	$9.4\times 10^{-11}\pm 4\times 10^{-12}$	3/7
	y = 1.1	–	0.096±0.005	0.350±0.010	1230±100	$8.5\times 10^{-12}\pm 7\times 10^{-13}$	4/7
	y = 1.3	–	0.093±0.005	0.330±0.009	1200±100	$7.5\times 10^{-13}\pm 6\times 10^{-14}$	5/7
	y = 1.5	–	0.089±0.004	0.320±0.010	1200±80	$6\times 10^{-14}\pm 5\times 10^{-15}$	6/7
	y = 1.7	–	0.084±0.004	0.208±0.010	1110±104	$1.6\times 10^{-14}\pm 6\times 10^{-15}$	11/7
	y = 1.9	–	0.080±0.005	0.290±0.012	1200±108	$4.45\times 10^{-16}\pm 4\times 10^{-17}$	19/6
Figure 4	y = 0.1	$K^{-}$	0.112±0.004	0.240±0.012	1350±127	$6\times 10^{-7}\pm 4\times 10^{-8}$	2/5
p-p	y = 0.3	–	0.110±0.005	0.240±0.010	1300±87	$5.7\times 10^{-8}\pm 5\times 10^{-9}$	1/4
40 GeV	y = 0.5	–	0.106±0.005	0.260±0.008	1200±109	$5\times 10^{-9}\pm 7\times 10^{-10}$	11/5
	y = 0.7	–	0.102±0.005	0.285±0.009	1200±110	$4\times 10^{-10}\pm 6\times 10^{-11}$	3/5
	y = 0.9	–	0.098±0.006	0.310±0.011	1260±120	$3.2\times 10^{-11}\pm 6\times 10^{-12}$	1/4
	y = 1.1	–	0.095±0.005	0.350±0.009	1235±100	$2.7\times 10^{-12}\pm 4\times 10^{-13}$	2/4
	y = 1.3	–	0.092±0.005	0.280±0.010	1205±87	$1.6\times 10^{-13}\pm 5\times 10^{-14}$	2/3
	y = 1.5	–	0.088±0.006	0.250±0.009	1200±94	$9.8\times 10^{-15}\pm 6\times 10^{-16}$	2/2
	y = 1.7	–	0.084±0.004	0.208±0.012	1100±150	$7\times 10^{-16}\pm 5\times 10^{-17}$	5/2
	y = 1.9	–	0.080±0.005	0.290±0.012	1200±108	$1.25\times 10^{-17}\pm 5\times 10^{-18}$	2.5/-
	y = 2.1	–	0.076±0.005	0.200±0.010	1200±100	$1.7\times 10^{-18}\pm 4\times 10^{-19}$	0.1/-
Figure 3	y = 0.1	$K^{+}$	0.121±0.005	0.240±0.010	1708±93	$1.1\times 10^{-6}\pm 6\times 10^{-7}$	1.5/5
p-p	y = 0.3	–	0.118±0.006	0.240±0.007	1600±68	$1.2\times 10^{-7}\pm 6\times 10^{-8}$	2/7
80 GeV	y = 0.5	–	0.114±0.005	0.280±0.011	1660±120	$1.1\times 10^{-8}\pm 4\times 10^{-9}$	1.5/7
	y = 0.7	–	0.110±0.005	0.320±0.010	1630±120	$1\times 10^{-9}\pm 6\times 10^{-10}$	1.5/6
	y = 0.9	–	0.107±0.005	0.290±0.012	1638±142	$9\times 10^{-11}\pm 4\times 10^{-12}$	2/5
	y = 1.1	–	0.105±0.006	0.300±0.009	1601±60	$8\times 10^{-12}\pm 6\times 10^{-13}$	2/7
	y = 1.3	–	0.100±0.006	0.272±0.008	1601±90	$6\times 10^{-14}\pm 7\times 10^{-15}$	8/7
	y = 1.5	–	0.096±0.005	0.305±0.010	1578±90	$5\times 10^{-15}\pm 5\times 10^{-16}$	2/7
	y = 1.7	–	0.093±0.006	0.300±0.010	1500±100	$5.1\times 10^{-15}\pm 5\times 10^{-16}$	4/6
	y = 1.9	–	0.090±0.005	0.250±0.011	1520±108	$3.5\times 10^{-16}\pm 6\times 10^{-17}$	4/6
Figure 4	y = 0.1	$K^{-}$	0.121±0.006	0.240±0.010	1700±115	$5.3\times 10^{-7}\pm 4\times 10^{-8}$	1.5/5
p-p	y = 0.3	–	0.118±0.006	0.240±0.007	1600±79	$6\times 10^{-8}\pm 4\times 10^{-9}$	1/4
80 GeV	y = 0.5	–	0.114±0.004	0.280±0.011	1660±123	$5.8\times 10^{-9}\pm 6\times 10^{-10}$	2/4
	y = 0.7	–	0.110±0.005	0.314±0.010	1630±120	$5\times 10^{-10}\pm 5\times 10^{-11}$	0.4/4
	y = 0.9	–	0.107±0.005	0.270±0.010	1638±142	$3.8\times 10^{-11}\pm 6\times 10^{-12}$	2/4
	y = 1.1	–	0.103±0.004	0.290±0.008	1621±120	$2.9\times 10^{-12}\pm 5\times 10^{-13}$	16/5
	y = 1.3	–	0.100±0.004	0.262±0.012	1607±95	$2.3\times 10^{-13}\pm 5\times 10^{-14}$	5/5
	y = 1.5	–	0.096±0.005	0.300±0.010	1578±98	$1.7\times 10^{-14}\pm 5\times 10^{-15}$	0.7/2
	y = 1.7	–	0.092±0.005	0.270±0.010	1535±110	$1.2\times 10^{-15}\pm 7\times 10^{-16}$	6.5/
	y = 1.9	–	0.089±0.004	0.250±0.010	1543±100	$5.6\times 10^{-17}\pm 5\times 10^{-18}$	2/-
	y = 2.1	–	0.085±0.006	0.200±0.012	1570±100	$6\times 10^{-19}\pm 6\times 10^{-20}$	0.2/-
Figure 3	y = 0.1	$K^{+}$	0.128±0.006	0.230±0.008	2130±120	$9\times 10^{-7}\pm 7\times 10^{-8}$	2/6
p-p	y = 0.3	–	0.126±0.005	0.250±0.011	2117±110	$9.1\times 10^{-8}\pm 7\times 10^{-9}$	1/6
158 GeV	y = 0.5	–	0.121±0.005	0.275±0.012	2110±110	$9.2\times 10^{-9}\pm 5\times 10^{-10}$	1/6
	y = 0.7	–	0.120±0.005	0.295±0.011	2025±120	$9\times 10^{-10}\pm 6\times 10^{-11}$	0.5/6
	y = 0.9	–	0.116±0.005	0.280±0.012	2000±115	$8.5\times 10^{-11}\pm 5\times 10^{-12}$	1/6
	y = 1.1	–	0.112±0.005	0.240±0.010	2070±130	$7\times 10^{-12}\pm 6\times 10^{-13}$	2/6
	y = 1.3	–	0.107±0.004	0.278±0.011	2050±112	$6\times 10^{-13}\pm 6\times 10^{-14}$	1/5
	y = 1.5	–	0.105±0.004	0.248±0.011	2020±103	$5\times 10^{-14}\pm 7\times 10^{-15}$	2/5
	y = 1.7	–	0.103±0.005	0.268±0.010	1980±120	$4.1\times 10^{-15}\pm 7\times 10^{-16}$	2/5
	y = 1.9	–	0.098±0.006	0.217±0.010	1966±106	$3\times 10^{-16}\pm 4\times 10^{-17}$	5.5/4
	y = 2.1	–	0.097±0.006	0.210±0.011	1993±100	$2\times 10^{-17}\pm 5\times 10^{-18}$	11/5
	y = 2.3	–	0.094±0.005	0.300±0.009	1960±100	$4\times 10^{-18}\pm 5\times 10^{-19}$	13/3
	y = 2.5	–	0.090±0.004	0.230±0.010	1976±90	$2\times 10^{-19}\pm 4\times 10^{-20}$	1.5/-
Figure 4	y = 0.1	$K^{-}$	0.128±0.005	0.230±0.009	2130±126	$6\times 10^{-7}\pm 6\times 10^{-8}$	1/6
p-p	y = 0.3	–	0.125±0.004	0.255±0.007	2130±100	$5.8\times 10^{-8}\pm 6\times 10^{-9}$	0.3/5
158 GeV	y = 0.5	–	0.122±0.006	0.275±0.010	2130±103	$6\times 10^{-9}\pm 6\times 10^{-9}$	0.5/6
	y = 0.7	–	0.118±0.004	0.295±0.012	2057±102	$5.4\times 10^{-10}\pm 4\times 10^{-11}$	1/5
	y = 0.9	–	0.114±0.006	0.280±0.012	2000±115	$4.5\times 10^{-11}\pm 7\times 10^{-12}$	1/7
	y = 1.1	–	0.110±0.006	0.230±0.008	2100±117	$3.7\times 10^{-12}\pm 6\times 10^{-13}$	3/7
	y = 1.3	–	0.106±0.006	0.270±0.008	2070±117	$3\times 10^{-13}\pm 5\times 10^{-14}$	11/6
	y = 1.5	–	0.104±0.005	0.238±0.008	2020±105	$2.3\times 10^{-14}\pm 5\times 10^{-15}$	2/6
	y = 1.7	–	0.101±0.004	0.268±0.011	1987±88	$1.7\times 10^{-15}\pm 6\times 10^{-16}$	30/4
	y = 1.9	–	0.097±0.006	0.217±0.009	1971±90	$1\times 10^{-16}\pm 5\times 10^{-17}$	1/3
	y = 2.1	–	0.094±0.005	0.210±0.009	1993±110	$7\times 10^{-18}\pm 5\times 10^{-19}$	37/2
	y = 2.3	–	0.092±0.005	0.290±0.010	1570±100	$4.1\times 10^{-19}\pm 4\times 10^{-20}$	2/1
	y = 2.5	–	0.089±0.004	0.230±0.008	1980±120	$6.8\times 10^{-20}\pm 6\times 10^{-21}$	4/-
Figure 5	y = 0.1	$\bar{p}$	0.120±0.006	0.089±0.009	800±56	$1.01\times 10^{-7}\pm 6\times 10^{-8}$	8/5
p-p	y = 0.3	–	0.117±0.005	0.100±0.008	800±70	$1.25\times 10^{-8}\pm 4\times 10^{-9}$	20/5
31 GeV	y = 0.5	–	0.113±0.004	0.060±0.008	800±93	$6.3\times 10^{-10}\pm 5\times 10^{-11}$	10/4
	y = 0.7	–	0.109±0.005	0.120±0.010	700±68	$5.6\times 10^{-11}\pm 4\times 10^{-12}$	12/3
	y = 0.9	–	0.060±0.007	0.120±0.007	755±110	$2.6\times 10^{-12}\pm 4\times 10^{-13}$	8/3
Figure 5	y = 0.1	$\bar{p}$	0.129±0.004	0.055±0.008	1100±120	$8.4\times 10^{-8}\pm 4\times 10^{-9}$	3/1
p-p	y = 0.3	–	0.125±0.006	0.150±0.009	1000±110	$9.8\times 10^{-9}\pm 6\times 10^{-10}$	22/5
40 GeV	y = 0.5	–	0.121±0.005	0.120±0.009	1060±88	$7.8\times 10^{-10}\pm 5\times 10^{-11}$	11/3
	y = 0.7	–	0.116±0.004	0.140±0.009	1040±80	$5\times 10^{-11}\pm 5\times 10^{-12}$	19/4
	y = 0.9	–	0.090±0.005	0.100±0.008	1000±80	$2.5\times 10^{-12}\pm 5\times 10^{-13}$	7/4
Figure 5	y = 0.1	$\bar{p}$	0.137±0.006	0.120±0.007	1500±160	$1.5\times 10^{-7}\pm 6\times 10^{-8}$	36/7
p-p	y = 0.3	–	0.133±0.005	0.120±0.007	1400±100	$1.5\times 10^{-8}\pm 6\times 10^{-9}$	10/7
80 GeV	y = 0.5	–	0.124±0.006	0.060±0.007	1426±93	$1.25\times 10^{-9}\pm 5\times 10^{-10}$	5/7
	y = 0.7	–	0.122±0.005	0.100±0.007	1450±120	$1\times 10^{-10}\pm 6\times 10^{-11}$	4/7
	y = 0.9	–	0.110±0.005	0.060±0.008	1410±100	$8\times 10^{-12}\pm 5\times 10^{-13}$	12/6
	y = 1.1	–	0.107±0.005	0.120±0.009	1370±100	$4.8\times 10^{-13}\pm 6\times 10^{-14}$	10/6
	y = 1.3	–	0.102±0.006	0.120±0.010	1300±80	$3.3\times 10^{-14}\pm 4\times 10^{-15}$	14/5
	y = 1.5	–	0.086±0.006	0.290±0.011	1330±110	$6\times 10^{-16}\pm 6\times 10^{-17}$	277/4
Figure 5	y = 0.1	$\bar{p}$	0.144±0.005	0.060±0.006	1850±110	$2.3\times 10^{-7}\pm 4\times 10^{-8}$	4.5/8
p-p	y = 0.3	–	0.139±0.006	0.100±0.007	1730±120	$2.3\times 10^{-8}\pm 5\times 10^{-9}$	107/8
158 GeV	y = 0.5	–	0.134±0.005	0.100±0.008	1700±85	$2.2\times 10^{-9}\pm 4\times 10^{-10}$	7/8
	y = 0.7	–	0.130±0.006	0.160±0.011	1647±110	$2.2\times 10^{-10}\pm 6\times 10^{-11}$	3/7
	y = 0.9	–	0.121±0.006	0.120±0.009	1670±120	$1.65\times 10^{-11}\pm 6\times 10^{-12}$	3/6
	y = 1.1	–	0.117±0.004	0.120±0.007	1600±120	$1.3\times 10^{-12}\pm 5\times 10^{-13}$	2/5
	y = 1.3	–	0.113±0.005	0.150±0.011	1650±130	$1\times 10^{-13}\pm 6\times 10^{-14}$	13/6
	y = 1.5	–	0.110±0.005	0.150±0.012	1610±100	$5.3\times 10^{-15}\pm 5\times 10^{-16}$	2/3
	y = 1.7	–	0.106±0.004	0.150±0.010	1635±110	$3.3\times 10^{-16}\pm 7\times 10^{-17}$	3/2
	y = 1.9	–	0.088±0.005	0.085±0.006	1630±100	$1.1\times 10^{-17}\pm 6\times 10^{-18}$	4/1

## Data Availability

The data used to support the findings of this study are included within the article and are cited at relevant places within the text as references.
